# Autistic traits and alcohol use in adolescents within the general population

**DOI:** 10.1007/s00787-022-01970-3

**Published:** 2022-03-23

**Authors:** Lisa J. Pijnenburg, Anais Kaplun, Lieuwe de Haan, Magdalena Janecka, Lauren Smith, Abraham Reichenberg, Tobias Banaschewski, Arun L. W. Bokde, Erin Burke Quinlan, Sylvane Desrivières, Antoine Grigis, Hugh Garavan, Penny Gowland, Andreas Heinz, Bernd Ittermann, Jean-Luc Martinot, Marie-Laure Paillère Martinot, Frauke Nees, Dimitri Papadopoulos Orfanos, Tomáš Paus, Luise Poustka, Sarah Hohmann, Sabina Millenet, Juliane H. Fröhner, Michael N. Smolka, Henrik Walter, Robert Whelan, Gunter Schumann, Eva Velthorst, IMAGEN Consortium

**Affiliations:** 1 GGZ Rivierduinen, Institute for Mental Health Care, Leiden, The Netherlands; 2 Department of Psychiatry, Academic Medical Center, University of Amsterdam, Meibergdreef 5, 1105 AZ Amsterdam, The Netherlands; 3 Department of Psychiatry, Icahn School of Medicine at Mount Sinai, New York City, NY USA; 4 Seaver Autism Center for Research and Treatment, New York City, NY USA; 5 Department of Child and Adolescent Psychiatry and Psychotherapy, Central Institute of Mental Health, Medical Faculty Mannheim, Heidelberg University, Square J5, 68159 Mannheim, Germany; 6 Discipline of Psychiatry, School of Medicine and Trinity College Institute of Neuroscience, Trinity College Dublin, Dublin, Ireland; 7 Medical Research Council - Social, Genetic and Developmental Psychiatry Centre, Institute of Psychiatry, Psychology and Neuroscience, King’s College London, London, UK; 8 NeuroSpin, CEA, Université Paris-Saclay, 91191 Gif-sur-Yvette, France; 9 Department of Psychiatry, University of Vermont, Burlington, VT 05405 USA; 10 Department of Psychology, University of Vermont, Burlington, VT 05405 USA; 11 Sir Peter Mansfield Imaging Centre School of Physics and Astronomy, University of Nottingham, University Park, NG UK; 12 Department of Psychiatry and Psychotherapy, Charité – Universitätsmedizin Berlin, corporate member of Freie Universität Berlin, Humboldt-Universität zu Berlin, and Berlin Institute of Health, Campus Charité Mitte, Charitéplatz 1, Berlin, Germany; 13 Physikalisch-Technische Bundesanstalt (PTB), Braunschweig, Germany; 14 Physikalisch-Technische Bundesanstalt (PTB), Berlin, Germany; 15 Maison de Solenn, Paris, France; 16 Institut National de la Santé et de la Recherche Médicale, INSERM Unit 1000 “Neuroimaging and Psychiatry”, UniversityParis Sud, University Paris Descartes, Sorbonne Université, Paris, France; 17 Department of Child and Adolescent Psychiatry, AP-HP, Pitié-Salpêtrière Hospital, Paris, France; 18 Institute of Cognitive and Clinical Neuroscience, Medical Faculty Mannheim, Central Institute of Mental Health, Heidelberg University, Square J5, Mannheim, Germany; 19 Department of Psychology, Bloorview Research Institute, Holland Bloorview Kids Rehabilitation Hospital, University of Toronto, Toronto, ON M6A 2E1 Canada; 20 Department of Psychiatry, Bloorview Research Institute, Holland Bloorview Kids Rehabilitation Hospital, University of Toronto, Toronto, ON M6A 2E1 Canada; 21 Department of Child and Adolescent Psychiatry and Psychotherapy, University Medical Centre Göttingen, von-Siebold-Str. 5, 37075 Göttingen, Germany; 22 Department of Psychiatry and Neuroimaging Center, Technische Universität Dresden, Dresden, Germany; 23 School of Psychology and Global Brain Health Institute, Trinity College Dublin, Dublin, Ireland; 24 PONS Research Group, Department of Psychiatry and Psychotherapy, Humboldt University, Campus Charite Mitte, Berlin, Germany; 25 Institute for Science and Technology of Brain-Inspired Intelligence (ISTBI), Fudan University, Shanghai, People’s Republic of China

**Keywords:** Adolescence, Alcohol use, Autistic traits, Social preference

## Abstract

It has been suggested that autistic traits are associated with less frequent alcohol use in adolescence. Our study seeks to examine the relationship between autistic traits and alcohol use in a large adolescent population. Leveraging data from the IMAGEN cohort, including 2045 14-year-old adolescents that were followed-up to age 18, we selected items on social preference/skills and rigidity from different questionnaires. We used linear regression models to (1) test the effect of the sum scores on the prevalence of alcohol use (AUDIT-C) over time, (2) explore the relationship between autistic traits and alcohol use patterns, and (3) explore the specific effect of each autistic trait on alcohol use. Higher scores on the selected items were associated with trajectories of less alcohol use from the ages between 14 and 18 (*b* = − 0.030; CI 95% = − 0.042, − 0.017; *p* < 0.001). Among adolescents who used alcohol, those who reported more autistic traits were also drinking less per occasion than their peers and were less likely to engage in binge drinking. We found significant associations between alcohol use and social preference (*p* < 0.001), nervousness for new situations (*p* = 0.001), and detail orientation (*p* < 0.001). Autistic traits (social impairment, detail orientation, and anxiety) may buffer against alcohol use in adolescence.

**Supplementary Information:**

The online version contains supplementary material available at 10.1007/s00787-022-01970-3.

## Introduction

Autism spectrum disorder (ASD) is a complex neurodevelopmental disorder, affecting more than 6 in 1000 children in Europe [1]. The disorder is characterized by social impairments leading to less engagement in social interactions, abnormalities in verbal and nonverbal communication and restricted, stereotyped interests and behaviors that persist into adulthood [2]. Subthreshold levels of autistic traits are also found in the general population [3, 4]. This is shown by a number of instruments, including the self-assessment screening instrument called the ‘Autism-Spectrum Quotient’, which is used to measure autistic traits, regardless of a clinical ASD diagnosis [5]. A systematic review showed that individuals within the general population had an average score of 17 on the before mentioned Autism-Spectrum Quotient compared to an average of 35 in a clinical ASD population [6]. Furthermore, results from a twin study suggested that autistic traits [as measured by the Social Responsiveness Scale (SRS)] were continuously distributed in both boys and girls [7].

Importantly, while ASD and subclinical autistic traits have been associated with various functional impairments [8, 9], associated benefits have also been reported. For example, a recent study using a large nationally representative cohort of British twins (*n* = 7781) found that children who focus strongly on the subject of interest and display little concern about fitting in tend to have better academic performance than their peers [10]. Another study, comparing people with ASD with healthy controls on decision-making, found that individuals with higher levels of autistic traits were more likely to make consistent decisions [11].

Apart from the reported academic benefits, it has been suggested that autistic traits may also be associated with certain positive health behaviors, such as a reduced tendency to start drinking and/or abusing alcohol [12]. Supportive of the idea that autistic traits may act as a buffer against alcohol use is the finding of a recent large-scale study that examined comorbidity within ten psychiatric diagnostic groups: developmental disorders were the only type of disorders that were not associated with later substance use [13, 14]

One possible explanation of reduced alcohol intake among adolescents with autistic traits, like a more rigid thinking pattern, could be that, once they decide not to start using alcohol, they will not change their decision due to peer pressure. Alternatively, a reduced tendency to start drinking may also be due to impaired social skills, causing individuals with autistic traits to have more limited interactions with their (substance-abusing) peers [15] and less access to social settings that encourage drinking and alcohol intoxication [12]. Peer influence is an important risk factor for drinking in adolescence [16, 17], and less engagement in social situations may, therefore, be protective against alcohol use.

However, findings relating to alcohol use among the non-clinical samples have been inconsistent. For example, De Alwis et al. [18] found that adults with autistic traits were less likely to report drinking to intoxication, but were at elevated risk for developing alcohol dependence. Contrary to this finding, a twin-study using data from two Swedish samples (including adults and children) found that individuals with more autistic traits indicated more alcohol and substance abuse [19]. Alcohol use among individuals with autistic traits could be potentially attributed to ‘self-medication’ to lower anxiety. Indeed, it has been suggested that some persons with high-functioning autism intentionally drink alcohol to cope with anxiety, to maintain friendships and gain access to new relationships [20, 21]. Interestingly, while this theory has been put forward in relation to ASD [20, 21], this pattern is generally not seen in adolescents with (social) anxiety [22].

These inconsistent findings are potentially due, at least in part, to several methodological limitations of those earlier studies, such as crude measures of alcohol use [19] or a failure to account for potential comorbid Attention Deficit Hyperactivity Disorder (ADHD) symptomatology. Accounting for ADHD symptoms is important because ADHD and its subclinical symptoms co-occur frequently with both autistic traits [19, 23, 24] and clinical ASD [9] and greatly influence alcohol use in this group. Indeed, in contrast to individuals with ASD traits alone, individuals with comorbid ADHD traits have been found to have an increased risk of alcohol abuse [14, 18, 25, 26]. It has been suggested that the underlying reasons for the increased alcohol consumption among youngsters with co-occurring ASD and ADHD symptoms could be in part genetic but may also be a direct, impulsive, response to dealing with stressors in the immediate environment [27, 28]. Autistic traits have been associated with an enhanced risk of exposure to environmental stress, e.g. to bullying or child abuse [27, 28]. The co-occurring (impulsive) traits associated with ADHD may cause the young individual to initiate alcohol use.

Understanding the relationship between autistic traits and alcohol use in adolescents may provide us with new insights into factors that may facilitate or prevent the development of alcohol use during adolescence, and may provide important clues about potential targets for interventions aimed at the reduction of alcohol use in adolescents.

Adolescence is a crucial, dynamic developmental period [29] and there is large heterogeneity in developmental patterns across individuals. Trajectories of alcohol use behaviors may provide more insight into what makes adolescents decide to start drinking and who is more likely to engage in unsafe drinking practices (like binge drinking) than cross-sectional data alone.

Using comprehensive data from the unique large-scaled IMAGEN cohort [30] including over 2400 young adolescents, we (1) examined the association between autistic traits relating to social skills and rigidity and differences in alcohol use between the ages of 14 and 18, (2) explored the association between these autistic traits and the amount of drinks per occasion and risk of binge drinking, and (3) explored whether there was a differential association between alcohol use and specific autistic traits.

## Methods

### Participants

The IMAGEN study is a multi-site multi-national longitudinal research project that includes data on 2462 14-year-old adolescents and their parents. Participants were recruited through high schools in eight European sites across the United Kingdom, Ireland, France, and Germany. Clinical and behavioral data were collected at baseline, and assessments were repeated at 2 (age 16) and 4 years (age 18) follow-up. A detailed description of recruitment and research procedures has been published elsewhere [30]. The study protocol was approved by the King’s College London (KCL) College Research Ethics Committee CREC/06/07-71 and each study site was granted approval from the local research ethics committee. Written consent was obtained from each participant and his or her guardian.

### Instruments

#### Social preference and rigidity

To assess autistic traits in the IMAGEN population, items were derived from the following scales designed to capture personality traits and behavior in non-clinical populations:

The Neuroticism, Extraversion, Openness Personality Five Factor Inventory (NEO-FFI) [31] contains 60 questions on separate personality traits organized by the five-factor model (neuroticism, extraversion, openness to experience, agreeableness and conscientiousness). The items are measured on a five-point Likert scale ranging from ‘strongly agree’ to ‘strongly disagree’. We selected three items from this scale: ‘I like to have a lot of people around me’, ‘I usually prefer to do things alone’ and ‘I really enjoy talking to people’.

The Temperament and Character Inventory Questionnaire (TCI) [32] is a self-report instrument that assesses four temperament (novelty seeking, harm avoidance, reward dependence, and persistence) and three character (self-directness, cooperativeness and self-transcendence) dimensions. It comprises 240 items rated on a five-point Likert scale, ranging from ‘definitely true’ to ‘definitely false’. Two item were selected from the TCI: ‘I hate to change the way I do things, even if many people tell me there is a new and better way to do it’ and ‘I like to pay close attention to details in everything I do’.

The Strengths and Difficulties Questionnaire (SDQ) [33, 34] assesses peer relations, conduct problems, emotional problems, prosocial behaviors and hyperactivity using 25 items on a 3- point Likert scale, ranging from ‘true’ to ‘not true’. From this scale we selected two items: ‘I have one good friend or more’ and ‘I am nervous in new situations. I easily lose confidence’.

When necessary, item scores were reversed, so that for each item a higher score represented more severe traits (Table 1).

**Table 1:** Selected questions on autistic traits

Question	Item name
NEO-FFI:	
-I like to have a lot of people around me.*	People
-*I usually prefer to do things alone*	Alone
-I really enjoy talking to people.*	Talking
TCI:	
-*I hate to change the way I do things, even if many people tell me there is a new and better way to do it*	Change
-*I like to pay close attention to details in everything I do*	Details
SDQ	
-I have one good friend or more.*	Friends
-*I am nervous in new situations. I easily lose confidence*	Nervous

*NEO-FFI* Neuroticism, Extraversion, Openness Personality Inventory Five Factor Inventory, *TCI* Temperament and Character Inventory Questionnaire, *SDQ* Strengths and Difficulties Questionnaire

*Lower scores on this item translate to higher scores

### ASD and ADHD diagnoses

IMAGEN is a general cohort study, which means participants with a clinical diagnosis of ASD or ADHD were excluded. However, some participants may still have met the diagnostic criteria for ASD or ADHD. We used the Development and Well-being Assessment (DAWBA) to identify and exclude these individuals from analyses. The DAWBA includes a package of interviews, questionnaires and rating techniques designed to diagnose psychiatric problems in 2–17 year-olds. Clinical raters use responses from informants to assign ICD-10 and DSM-IV psychiatric diagnoses. These clinician-rated DAWBA diagnoses are considered reliable and are able to discriminate well between community and clinical samples and between different diagnoses [35].

### Alcohol use

To measure alcohol use, we used a short version of the Alcohol Use Disorder Identification Test (AUDIT), called the AUDIT-Consumption (AUDIT-C [36]). The items were designed to measure (1) frequency of alcohol use (*How often do you have a drink containing alcohol?)*, (2) the amount of alcohol used (*How many standard drinks containing alcohol do you have on a typical day when drinking?),* and (3) the frequency of binge drinking (*How often do you have six or more drinks on one occasion?*). The AUDIT-C is a reliable measure of hazardous alcohol use [36] and has been validated in adolescent populations [37]. Questions are rated on a five-point Likert scale, ranging from 0 to 4 with higher scores indicating more frequent and more severe alcohol use (Table S1). The cut-off scores for alcohol use disorder in adults are  ≥ 3 (women) or  ≥ 4 (men) [36]. For adolescents, an item sum score of 2 or higher has been considered ‘problematic alcohol use’ [37].

### Intelligence Quotient (IQ)

IQ was estimated with the WISC-IV subtests Vocabulary (crystalized IQ marker) and Matrix Reasoning (fluid IQ marker) [38, 39]. See supplemental Table S2 for a description of the WISC-IV.

### Statistical analyses

#### Preparatory analyses

Item selection to measure autistic traits was performed using a stepwise approach. First, we evaluated all items from the SDQ, NEO-FFI, and TCI, using the DSM-IV ASD classification as reference [40]. This resulted in 18 potentially relevant questions. Second, we ran a principle component analysis to explore which items loaded best on the factors corresponding to the ASD DSM-IV classification (see supplementary material Table S4 for principle component analysis including the final selected items). Several items were removed because they loaded on multiple factors or did not load well on any of the factors (< 0.3). We checked the inter-correlation of the seven included autistic trait variables using Pearson correlation coefficients. Distribution of the social preference/skills and rigidity domain scores was also explored visually with the aid of a histogram. Lastly, we examined whether individuals with a suspected clinical ASD diagnosis (defined as DAWBA clinical ASD rating of > 0) had higher cumulative scores on the selected questions compared to those without. Individuals with a suspected ASD diagnosis were excluded from further analyses.

### Association between autistic traits at age 14 and alcohol use in adolescents age 14–18

To explore the association between the autistic trait scores and alcohol use over time, we first looked at AUDIT-C scores at age 14, 16, and 18. Next, individual intercept and slope coefficients for the individual AUDIT-C scores over time were calculated for all subjects. The intercept refers to the AUDIT-C scores at age 14 and the slope shows the change of the AUDIT-C scores over time. An increase in AUDIT-C score, reflected by the slope coefficient, refers to an increase in alcohol use.

To test if drinking behaviors at baseline and their change over time were influenced by the cumulative score of the autistic trait domains, we used linear regression models including AUDIT-C intercept and slope coefficients as dependent variables and sum autistic trait score as independent variable, controlling for sex, site, presence of clinical DAWBA ratings of ADHD and IQ. We then examined the association between the social preference/skills and rigidity scores and amount of drinks per occasion in participants who indicated they were drinking at least once a month, using linear regression models. Lastly, we aimed to examine the differential influence of specific types of autistic traits on alcohol use by exploring the association between individual items and intercept and slope coefficients of the AUDIT-C scores.

All analyses were performed in Stata/MP 14.2 (StataCorp, College Station, TX) [41]. Statistical significance was determined as a *p* value < 0.006 (0.05/8) due to eight statistical tests done on the dependent variable.

## Results

### Sample description

Out of 2462 IMAGEN participants, the results of the DAWBA pointed to four individuals with clinically severe ASD symptoms, who were excluded from further analyses. Another 413 had insufficient data available on questions of the autistic trait domains or ASD diagnosis, retaining a sample of 2045 participants for the main analyses (see Table 2 for their demographic characteristics).

**Table 2:** Sample characteristics

	Study sample *(n* = *2045)*
Ratio male/female; *n (%)*	1001 (49.0%)/1044 (51.0%)
Age at baseline; *M* (SD)	14.45 (0.408)
*Participation rate per center, n (%)*	
London	246 (12.6%)
Nottingham	302 (15.5%)
Dublin	179 (9.2%)
Berlin	244 (12.5%)
Hamburg	253 (12.9%)
Mannheim	230 (11.8%)
Paris	250 (12.8)
Dresden	251 (12.8%)
*Measures:*	
IQ; *M (SD)*	38.25 (4.97)
AUDIT-Consumption^a^; *M (SD)*	
Age 14	1.08 (1.53)
Age 16	2.87 (2.32)
Age 18	4.23 (2.53)
Sum score autistic trait items at age 14; *M (SD)*	17.03 (3.35)

^a^Alcohol Use Disorder Identification Test—Consumption (AUDIT-C)

The cumulative scores of the autistic trait domains were normally distributed (Fig. S1). Pearson correlation coefficients indicated weak, non-significant, correlations between measures underlying the autistic trait domains (Table S3). Participants with a clinical DAWBA rating of ASD (*n* = 4) had significantly higher autistic trait sum scores compared to the rest of the sample (mean of 21.00 vs 17.03, *t* = 2.37, *p* = 0.018). The percentage of participants in the sample that reported using alcohol at least once a month increased with age: 52% at age 14, 83% at age 16 and 93% at age 18.

### Autistic traits and trajectories of alcohol use

**Fig. 1:**
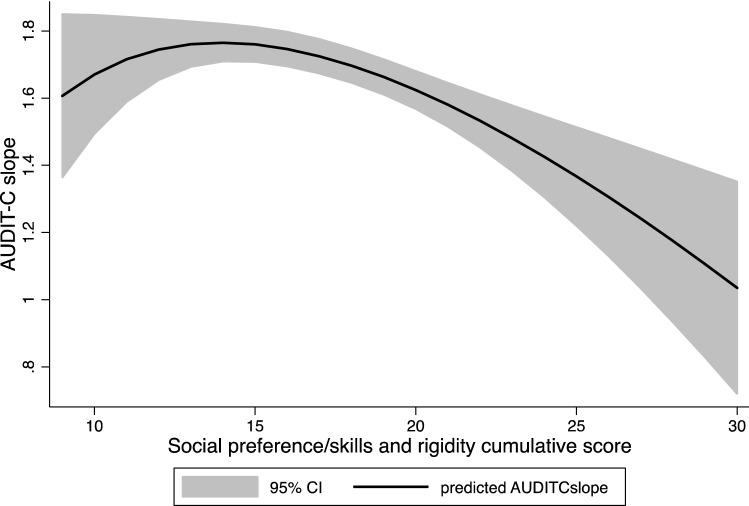
Association between AUDIT-C slope and item sum score

Cumulative scores on the selected questions of the social preference/skills and rigidity domains did not predict drinking behavior at age 14 (*b* = − 0.024; CI 95% = − 0.045, − 0.004; *p* = 0.021), but individuals with a higher total score engaged in drinking behavior less frequently between ages 14 and 18 (*b* = − 0.030; CI 95% = − 0.042, − 0.017; *p* < 0.001). Girls showed a smaller increase in alcohol use between ages 14 and 18 than boys (*b* = − 0.651; CI 95% = − 0.735, − 0.568; *p* < 0.001), but the autistic trait domains showed a similar predictive effect in both boys and girls. Interestingly, not correcting for ADHD did not alter the results. Using ‘country’ instead of ‘center’ as a variable gave similar results. See Fig. 1 for the association between the slope coefficients of the total AUDIT-C scores and the SPAR total scores and supplemental table (Table S3) for the associations between the intercepts and slopes of the AUDIT-C and the autistic traits divided by sex.

### Association between autistic traits and the amount and frequency of alcohol use

Table 3 presents results of the regression analyses on social preference/skills and rigidity total scores and AUDIT-C scores at follow-up in participants that consumed alcohol monthly or less. Regression analyses per time-point among those participants showed that higher autistic trait sum scores were associated with fewer drinks consumed per occasion at age 18 (*b* = − 0.024; CI 95% = − 0.041, − 0.007; *p* = 0.005); as well as less binge drinking at age 14 (*b* = − 0.020; CI 95% = − 0.033, − 0.008; *p* =  0.002), age 16 (*b* = − 0.025; CI 95% = − 0.039, − 0.011; *p* = 0.001) and age 18 (*b* = − 0.023; CI 95% = − 0.038, − 0.007; *p* = 0.004).

**Table 3:** Association between cumulative social preference/skills and rigidity scores and items 2 and 3 of AUDIT-C

	Coef.	CI 95%	Std. error	*β*	*t*	*p*	Adj. *R*^2^
*How many standard drinks containing alcohol do you have on a typical day when drinking?*^*a*^							
14 (*n* = 1078, 52%)	− 0.014	− 0.027, − 0.000	0.007	− 0.063	− 2.00	0.046	0.022
16 (*n* = 1370, 83%)	− 0.023	− 0.039, − 0.006	0.008	− 0.074	− 2.70	0.007	0.042
18 (*n* = 1363, 93%)	− 0.024	− 0.041, − 0.007	0.009	− 0.074	− 2.80	0.005*	0.130
*How often do you have six or more drinks on one occasion?*^*b*^							
14 (*n* = 1078, 52%)	− 0.020	− 0.033, − 0.008	0.006	− 0.100	− 3.14	0.002*	0.009
16 (*n* = 1370, 83%)	− 0.025	− 0.039, − 0.011	0.007	− 0.095	− 3.45	0.001*	0.034
18 (*n* = 1363, 93%)	− 0.023	− 0.038, − 0.007	0.008	− 0.076	− 2.89	0.004*	0.123

All measures have been controlled for sex, IQ, site and ADHD-ratings. Alcohol Use Disorder Identification Test—Consumption. Adj. *R*^2^: percentage of variance that is accounted for, 1 meaning 100%

*p < 0.006, significant

^a^Ranging from 1 (1 or 2) to 5 (10 or more)

^b^Ranging from 1 (never) to 5 (daily or almost daily)

### The association between different types of autistic traits and alcohol use

Our findings suggest that reduced social interest, more attention to detail and being nervous in new situations were associated with lower AUDIT-C scores (Table 4).

**Table 4:** Associations between social preference/skills and rigidity items and AUDIT-C score trajectories

	People	Alone	Talking	Change	Details	Friends	Nervous
AUDIT-C^a^							
Intercept	*b* = − 0.115 (− 0.198; − 0.033)^b^ *t* = − 2.74 *p* = 0.006	*b* = 0.067 (− 0.005, 0.140)^b^ *t* = 1.82 *p* = 0.068	*b* = 0.086 (− 0.011, 0.184)^b^ *t* = 1.73 *p* = 0.083	*b* = − 0.020 (− 0.093, 0.053)^b^ *t* = − 0.53 *p* = 0.594	*b* = − 0.033 (− 0.105, 0.039)^b^ *t* = − 0.89 *p* = 0.372	*b* = − 0.105 (− 0.206, − 0.005)^b^ *t* = − 2.06 *p* = − 0.040	*b* = − 0.074 (− 0.125, − 0.023)^b^ *t* = − 2.86 *p* = 0.004*
Slope	*b* = − 0.032 (− 0.082, 0.018)^b^ *t* = − 1.24 *p* = 0.213	*b* = − 0.076 (− 0.119, − 0.032)^b^ *t* = − 3.38 *p* = 0.001*	*b* = − 0.141 (− 0.200, − 0.082)^b^ *t* = − 4.68 *p* < 0.001*	*b* = 0.039 (− 0.005, 0.083)^b^ *t* = 1.74 *p* = 0.082	*b* = − 0.108 (− 0.152, − 0.064)^b^ *t* = − 4.83 *p* < 0.001*	*b* = 0.025 (− 0.036, 0.086)^b^ *t* = 0.80 *p* = 0.424	*b* = 0.050 (0.020, 0.081)^b^ *t* = 3.20 *p* = 0.001*

All measures have been controlled for sex, IQ, site and ADHD-ratings

*p < 0.006, significant

^a^Alcohol Use Disorder Identification Test—Consumption

^b^95% Confidence Interval

We observed a smaller increase in AUDIT-C scores over time among individuals who indicated that they preferred to do things alone (*b* = − 0.076; CI 95% = − 0.119, − 0.032; *p* = 0.001) and among those that did not enjoy talking to people (*b* = − 0.141; CI 95% = − 0.200, − 0.082; *p* < 0.001). However, ‘not having many friends’ was not associated with alcohol use at age 14 (*b* = − 0.105; CI 95% = − 0.206, − 0.005; *p* = 0.040).

In addition, higher scores on the items referring to stronger attention to detail (*b* = − 0.108; CI 95% = − 0.152, − 0.064; *p* < 0.001) and being nervous in new situations were associated with less alcohol use over time (*b* = 0.050; CI 95% = 0.020, 0.081; *p* = 0.001). Being nervous in new situations was also associated with a lower level of alcohol use at age 14 (*b* = 0.074; CI 95% = − 0.125, − 0.023; *p* = 0.004).

## Discussion

In a large sample of adolescents that were followed-up from ages 14–18, we sought to explore the relationship between autistic traits and alcohol use in adolescents. Our findings suggest that, after adjusting for suspected ADHD symptomatology, autistic traits are associated with less frequent use of alcohol. Individuals with higher total scores on questions relating to social preference/skills and rigidity were less likely to start using alcohol during adolescence and to binge drink compared to their peers. Specifically, adolescents with less social interest, higher levels of attention to details, and higher levels of nervousness in new situations, used alcohol less frequently.

Our findings support the hypothesis that reduced participation in social activities, and consequently, less exposure to substance using peers, may protect adolescents from drinking alcohol. There are two, not mutually exclusive, theories on why avoidance of social situations is a common trait in people with ASD. The first theory is the ‘social motivation theory’: people with autistic traits are believed to assign less reward values to social stimuli [42–45]. The second possible explanation for higher social avoidance could be higher levels of social anxiety. Experiencing anxiety in social situations is common for individuals with autistic traits [23, 46] and social avoidance could be the consequence of social anxiety. Not surprisingly, social anxiety in itself has been negatively associated with alcohol use among college students [47] and shown to be protective against alcohol use in early adolescence [22]. Future research is needed to be able to disentangle these two mechanisms.

In addition, we found an association between higher attention to detail and less alcohol use over time. There are two plausible hypotheses that may help explain the association between detail orientation and alcohol use in adolescence. The first hypothesis suggests that alcoholic beverages are less appealing to people that rate high on detail orientation because this trait is thought to be related to higher sensory sensitivity [48], especially olfactory sensitivity. A stronger sense for the smell of alcohol might negatively influence the taste of alcoholic drinks and, therefore, discourage adolescents to start drinking. Future research is needed to explore if autistic traits indeed alter the taste and smell of alcoholic consumptions in such a way that it negatively influences the adolescent’s desire to start drinking alcohol. A second hypothesis suggests a direct negative effect of higher levels of detail orientation on social functioning. That is, higher levels of detail orientation reduce the ability to see the ‘big picture’, or integrate information to a coherent whole [11, 49]. To navigate through complex social situations with many implicit rules and subtle cues, integration of many sources of information is essential. Failure to integrate all this information in a context might contribute to everyday social difficulties [50] and increase social impairment and avoidance of social situations, including those where alcohol is used.

Lastly, we explored items related to nervousness for new situations. Interestingly, participants that were nervous in new situations were drinking less at age 14 and were less likely to start drinking compared to their peers. This finding is contradicts the idea that alcohol is commonly used as a regulator of negative emotions [51] at least in young adolescents. If participants that indicated nervousness in new situations would use alcohol as self-medication to reduce their anxiety, we would have expected to see an equal or more substantial progression in alcohol use once they started drinking.

Despite the social aspects of alcohol consumption, there is one important aspect that contributes to starting to drink alcohol in adolescence that needs to be addressed: drinking under the age of 18 is forbidden by law in almost all countries included in this study (with the exception of Germany, where the age limit for drinking beer is 16). In other words, to be able to drink alcohol, some form of disobedient behavior is required; for example, lying about ones age to purchase alcohol. For people with autistic traits this poses a possible challenge, because the ability to lie or cheat is suggested to be reduced in ASD [52]. More rigid definitions of ‘bad behavior’ and higher levels of rule-orientation [53] may withhold adolescents with autistic traits from drinking alcohol. However, this hypothesis should be explored by future research. Future research should also consider other societal influences and differences. Fr example, the tendency to avoid social situations, and thus minimize exposure to alcohol use, may vary across more or less individualistic society (where such social avoidance is more or less permitted).

### Strengths and limitations

The results of this study must be interpreted in the light of some limitations. First, we selected items from three different scales and some questions may have merely revealed personality (e.g. low extraversion and novelty seeking) rather than autistic traits solely. Also, our social preference/skills and rigidity domains do not cover the full spectrum of autistic traits and parallels with autistic traits and ASD must, therefore, be considered with caution. However, there are also multiple factors that support the use of the selected items. First, we did select questions that were similar to those probed for by the DSM-IV [40] criteria and the cumulative score gives a broader picture of autistic traits and alcohol use in adolescents than when only social preference items were used. The questions we used were largely uncorrelated, which supports the idea that they measured unique features. Furthermore, we found that participants with a clinical DAWBA rating of ASD showed a significantly higher combined score within the autistic traits items that were selected than the participants without an ASD diagnosis.

Second, although the associations between autistic traits and alcohol use we found were significant, effect sizes were small. Nevertheless, the effects we found were consistent across different types of traits, suggesting a non-negligible association between a broader ASD like phenotype and reduced alcohol use in adolescence.

Third, self-report measures may be unreliable when it comes to illicit behavior like underage drinking. However, although the AUDIT-C is a self-report instrument, it has been validated in both adult an adolescent populations and considered the best available self-report method for measuring alcohol use [36]. In addition, there is no reason to believe that such under-reporting bias would influence answers of people with higher autistic traits differently than those of the rest of the participants. If anything, given the suggested reduced ability to lie in ASD, participants with higher cumulative scores may be more honest about their drinking behavior. On the other hand, we cannot rule out that adolescents with lower autistic trait scores may have falsely exaggerated their alcohol consumption in an attempt to impress the researcher, although this is unlikely given the private nature of the assessments. The strength of this study lies in the large sample size with a relatively long follow-up, during an important developmental period.

In sum, adolescence is a crucial period to experiment with alcohol and to learn about the consequences of consuming alcohol. Using a validated scale to measure alcohol consumption (AUDIT-C) this study provides important insights into factors influencing trajectories of alcohol use in adolescents, which can inspire future preventive interventions aimed at preventing alcohol use. Specifically, our findings suggest that preventive training focused on social dynamics, including vulnerability of peer pressure and changing personal decisions in groups, could be effective in minimizing risk for future alcohol use.

## Supplementary Information

Below is the link to the electronic supplementary material.

Supplementary file1 (DOCX 65 KB)
