# Supplementary material for: Autistic traits and alcohol use in adolescents within the general population
Source: Eur Child Adolesc Psychiatry. 2022 Mar 23;32(9):1633–42. doi: 10.1007/s00787-022-01970-3 (PMC10460309; doi:10.1007/s00787-022-01970-3)
Supplement: Supplementary file 1 — Supplementary file1 (DOCX 65 KB) [file 787_2022_1970_MOESM1_ESM.docx]

Supplement

**Autistic traits and alcohol use in adolescents within the general population**

European Child & Adolescent Psychiatry

L. J. Pijnenburg, M.D.

l.j.pijnenburg@amsterdamumc.nl

*Table S1: Alcohol Use Disorder Identification Test - Consumption*

| **AUDIT-C** | |
| --- | --- |
| **Question** | **Scale** |
| 1. *How often do you have a drink containing alcohol?* | 0 *(never)*  1 *(monthly or less)*  2 *(2-4 times a month)*  3 *(2-3 times a week)*  4 *(4 or more times a week)* |
| 1. *How many standard drinks containing alcohol do you have on a typical day when drinking?* | 0 (*1 or 2*)  1 *(3 or 4)*  2 *(5 or 6)*  3 *(7 to 9)*  4 (*10 or more*) |
| 1. *How often do you have six or more drinks on one occasion?* | 0 (*never*)  1 *(less than monthly)*  2 *(monthly)*  3 *(weekly)*  4 (*daily or almost daily*) |

﻿

Table S2: *Wechsler Intelligence Scale for Children—Fourth Edition (WISC-IV)*

WISC is an individually administered test battery that assesses intelligence in school aged children (6–16 years, 11 months). The 4th edition (Wechsler 2003) comprises 10 core subtests, yielding four index scores that combine into one IQ score (Nader, Courchesne, Dawson, & Soulières, 2016).

| **WISC-IV subtests and index structure** | | | |
| --- | --- | --- | --- |
| Verbal Comprehension Index (VCI) | Perceptual Reasoning Index (PRI) | Working Memory index (WMI) | Processing Speed Index (PSI) |
| 1. Similarities | 1. Block design | 7. Digit span | 9. Coding |
| 1. Vocabulary | 1. Picture concepts | 8. Letter-number sequencing | 10. Symbol search |
| 1. Comprehension | 1. Matrix reasoning |  |  |

*FigS1:*

*Table S3: correlations between selected autistic trait items*

|  | **People** | **Alone** | **Talking** | **Change** | **Details** | **Friend** | **Nervous** |
| --- | --- | --- | --- | --- | --- | --- | --- |
| **People** | 1.0000 |  |  |  |  |  |  |
| **Alone** | 0.2970 | 1.0000 |  |  |  |  |  |
| **Talking** | 0.4022 | 0.2744 | 1.0000 |  |  |  |  |
| **Change** | 0.0371 | 0.1393 | 0.0794 | 1.0000 |  |  |  |
| **Details** | 0.0114 | 0.1253 | -0.0187 | 0.0724 | 1.0000 |  |  |
| **Friend** | 0.0876 | 0.0890 | 0.0706 | 0.0434 | 0.0245 | 1.0000 |  |
| **Nervous** | 0.1144 | 0.1296 | 0.1439 | 0.1202 | -0.0201 | 0.0778 | 1.0000 |

*Table S4: principle component analysis for selected autistic trait items*

|  | **Social preference** | **Rigidity** |
| --- | --- | --- |
| **People** | 0.5279 |  |
| **Alone** | 0.5001 |  |
| **Talking** | 0.5293 |  |
| **Change** |  | 0.5434 |
| **Details** |  | 0.6999 |
| **Friend** | 0.1989 |  |
| **Nervous** | 0.3021 |  |

*Table S5: Association between autistic trait item sum score and intercepts and slope coefficients of AUDIT-C^†^ scores divided per sex*

|  | **Coef. (C. I. 95%)** | | **Std. Error** | | **t** | | **p** | | **β** | |
| --- | --- | --- | --- | --- | --- | --- | --- | --- | --- | --- |
|  | ♂ | ♀ | ♂ | ♀ | ♂ | ♀ | ♂ | ♀ | ♂ | ♀ |
| **AUDIT-C***^†^* |  |  |  |  |  |  |  |  |  |  |
| Intercept | -0.019  (-0.049, 0.012) | -0.029  (-0.057, -0.002) | 0.016 | 0.014 | -1.18 | -2.10 | 0.237 | 0.036 | -0.024 | -0.041 |
| Slope | -0.040  (-0.060, -0.019) | -0.023  (-0.038, -0.007) | 0.010 | 0.008 | -3.84 | -2.88 | **<0.001** | **0.004** | -0.078 | -0.055 |

All measures have been controlled for IQ, site and ADHD-ratings.
* p < 0.006, significant
^†^ Alcohol Use Disorder Identification Test – Consumption (AUDIT-C)
